# BET Inhibitor JQ1 Attenuates Atrial Fibrillation Through Modulation of Fibrosis, Calcium Homeostasis, and Mitochondrial Function in a Murine Model

**DOI:** 10.3390/ijms262110363

**Published:** 2025-10-24

**Authors:** Zonghu Song, Nobuyuki Murakoshi, Dongzhu Xu, Binyang Xi, Yoshiko Murakata, Kazuhiro Aonuma, Kazuko Tajiri, Tomoko Ishizu

**Affiliations:** 1 Graduate School of Comprehensive Human Sciences, University of Tsukuba, 1-1-1 Tennodai, Tsukuba 305-8575, Ibaraki, Japan; s2030407@u.tsukuba.ac.jp (Z.S.); s2330449@u.tsukuba.ac.jp (B.X.); murakata@niid.go.jp (Y.M.); 2 Department of Cardiology, Faculty of Medicine, University of Tsukuba, 1-1-1 Tennodai, Tsukuba 305-8575, Ibaraki, Japan; xu_dongzhu@md.tsukuba.ac.jp (D.X.); tomoco@md.tsukuba.ac.jp (T.I.); 3 Life Science Innovation, University of Tsukuba, 1-1-1 Tennodai, Tsukuba 305-8575, Ibaraki, Japan; 4 Krannert Cardiovascular Research Center, Indiana University School of Medicine, 1801 North Senate Boulevard, Indianapolis, IN 46202, USA; kaonuma@iu.edu; 5 Department of Cardiology, National Cancer Center Hospital East, 6-5-1 Kashiwanoha, Kashiwa 277-8577, Chiba, Japan; ktajiri@east.ncc.go.jp

**Keywords:** atrial fibrillation, bromodomain, BET proteins, JQ1, fibrosis, calcium homeostasis, Sirt1, mitochondrial respiration, angiotensin II

## Abstract

Bromodomain and extraterminal domain (BET) proteins act as epigenetic regulators of gene transcription. BET inhibitors have shown therapeutic potential in various models of heart failure; however, their efficacy in atrial fibrillation (AF) remains incompletely understood. This study investigated the effects of the BET inhibitor JQ1 in a mice model of AF. Wild-type male C57BL/6 mice were randomized into four groups: control, JQ1 alone (50 mg/kg, intraperitoneal), angiotensin II (AngII; 1 μg/kg/min), and AngII plus JQ1. After 2 weeks, electrophysiological studies revealed that JQ1 significantly reduced AngII-induced AF inducibility and duration. It also attenuated left atrial enlargement, diastolic dysfunction, and cardiac fibrosis. Molecular analyses indicated that JQ1 suppressed the AngII-induced upregulation of pro-fibrotic genes and restored Sirt1 expression. Moreover, JQ1 also inhibited AngII-enhanced oxidized CaMKII and phosphorylated RyR2 levels. In HL-1 atrial cardiomyocytes, JQ1 improved calcium handling abnormalities, shortened prolonged action potential duration (APD), and restored mitochondrial respiration and adenosine triphosphate (ATP) production, all of which had been impaired by AngII. These findings suggest that BET inhibition by JQ1 mitigates structural and electrical remodeling associated with AF by attenuating atrial fibrosis, and by restoring calcium homeostasis, mitochondrial function, and Sirt1 expression. JQ1 may represent a novel therapeutic strategy for the prevention and treatment of AF.

## 1. Introduction

AF is the most common sustained cardiac arrhythmia encountered in clinical practice and is associated with significant morbidity and mortality. The global burden of AF has been steadily increasing and is projected to rise further in the coming years [1]. Although pulmonary vein isolation is currently recommended as a first-line therapy for patients with symptomatic AF, effective preventive strategies remain limited [2]. Conventional antiarrhythmic drugs targeting ion channels offer partial efficacy in select patients but are often limited by their proarrhythmic risks and adverse side-effect profiles. As such, the development of upstream preventive therapies targeting the underlying molecular and structural remodeling processes in AF remains an unmet clinical need [3].

BET proteins—including BRD2 (bromodomain containing protein 2), BRD3, BRD4, and BRDT (bromodomain testis-specific protein)—are epigenetic readers that recognize acetylated lysine residues on histone tails via tandem bromodomains (BD1 and BD2). In addition to their interactions with histones, BET proteins also bind non-histone proteins through various protein–protein interaction domains [4]. Primarily localized in the nucleus, BET proteins regulate gene transcription by recruiting transcriptional machinery to acetylated regions of chromatin. BET inhibitors competitively bind to the acetyl-lysine recognition pocket, displacing BET proteins from chromatin and altering gene expression patterns [5]. Emerging evidence has implicated BET proteins in the pathogenesis of diverse cardiovascular conditions [6]. JQ1, a selective small-molecule inhibitor of BET proteins, has been shown to improve cardiac function, suppress arrhythmias, reduce fibrosis and apoptosis, and extend survival in a mouse cardiomyopathy model of myocyte-specific *Lmna* deletion [7]. Furthermore, JQ1 attenuated cardiac fibrosis and preserved left ventricular (LV) function in mice subjected to transverse aortic constriction (TAC) [8,9], and exhibited cardioprotective effects even in advanced models of heart failure, including post-myocardial infarction [10]. Despite increasing evidence that BET proteins play a key role in the pathogenesis of cardiovascular diseases, their involvement in AF pathophysiology remains poorly understood. In this study, we aimed to evaluate the effects of the BET inhibitor JQ1 on atrial electrical and structural remodeling in a mouse model of AF, and to explore the underlying molecular mechanisms.

## 2. Results

### 2.1. JQ1 Treatment Inhibits AngII-Induced AF Susceptibility in Mice

**Figure 1:**
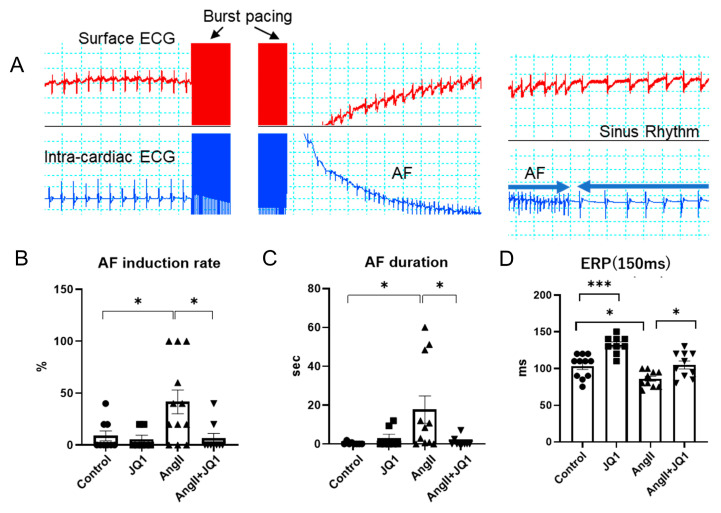
Electrophysiological assessment of AF susceptibility in mice. (**A**) Representative surface and intracardiac electrograms showing burst pacing–induced AF. (**B**) Summary of AF induction rates. (**C**) Duration of induced AF episodes. (**D**) Atrial effective refractory period (ERP) at a basic cycle length of 150 ms. Data are presented as mean ± SEM (*n* = 7–12 mice per group). * *p* < 0.05, *** *p* < 0.0005.

To investigate the effect of JQ1 on AF susceptibility, AF induction study was performed using transvenous catheter. After 30 seconds of burst pacing, AF was induced and spontaneously terminated (Figure 1A). AngII treatment significantly increased both AF inducibility and duration compared to the control group (Figure 1B,C). JQ1 administration markedly suppressed these AngII-induced effects. Atrial effective refractory period (AERP) was measured at a basic cycle length of 150 ms. AERP was significantly shortened in the AngII group compared to controls, whereas JQ1 treatment restored AERP to near-baseline levels (Figure 1D). These findings indicate that JQ1 reduces AF vulnerability by preventing AngII-induced electrophysiological remodeling, including AERP shortening. 

### 2.2. JQ1 Prevents AngII-Induced Atrial Enlargement and Diastolic Dysfunction

**Figure 2:**
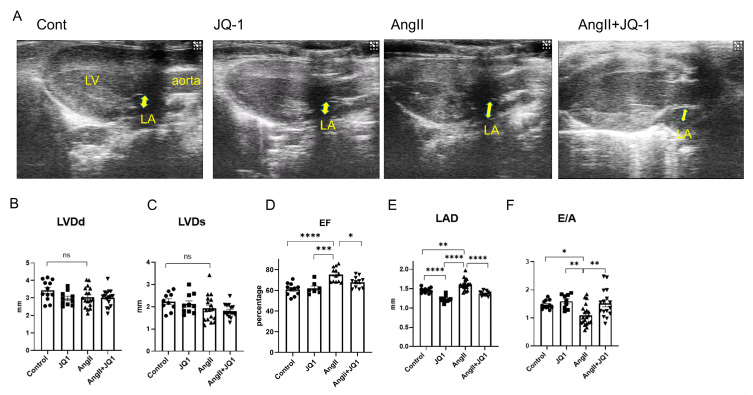
Cardiac structural and functional changes assessed by echocardiography. (**A**) Representative B-mode echocardiographic images. (**B**–**F**) Quantification of echocardiographic parameters: (**B**) LVDd; (**C**) LVDs; (**D**) EF; (**E**) LAD; and (**F**) E/A. Values are presented as mean ± SEM (*n* = 10–20 mice per group). * *p* < 0.05, ** *p* < 0.005, *** *p* < 0.0005, **** *p* < 0.0001.

Left atrial (LA) enlargement and diastolic dysfunction are established contributors to AF pathogenesis. Transthoracic echocardiography performed after two weeks of treatment revealed that Ang II induced left ventricular hypertrophy; however, neither Ang II nor JQ1 treatment appear to have effect on left ventricular dimensions or systolic function (Figure 2A). Based on the measurements, no significant differences were observed among groups in left ventricular end-diastolic diameter (LVDd), end-systolic diameter (LVDs), or left ventricular ejection fraction (LVEF) (Figure 2B–D). In contrast, AngII significantly increased left atrial diameter (LAD), an effect that was attenuated by JQ1 treatment (Figure 2E). JQ1 also improved the early-to-atrial filling velocity ratio (E/A), indicating mitigation of AngII-induced diastolic dysfunction (Figure 2F). These results suggest that JQ1 ameliorated AngII-induced LA enlargement and diastolic dysfunction without affecting LV size or systolic function. 

### 2.3. JQ1 Inhibits AngII-Induced Cardiac Fibrosis 

**Figure 3:**
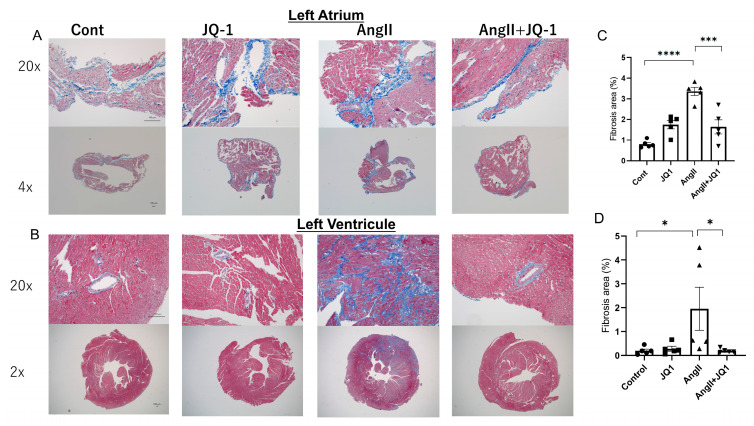
Histological analysis of cardiac fibrosis. (**A**,**B**) Representative Masson’s trichrome staining images of atrial and ventricular sections (blue = collagen). (**A**) Left atrium (upper: 20×, lower: 4×); (**B**) Left ventricle (upper: 20×, lower: 2×). (**C**,**D**) Quantification of fibrotic area as a percentage of total tissue area: (**C**) Left atrium; (**D**) Left ventricle. Data shown as mean ± SEM (*n* = 5 samples per group). * *p* < 0.05, *** *p* < 0.0005, **** *p* < 0.0001.

Masson’s trichrome staining demonstrated that AngII treatment increased fibrosis in both the left atrium and left ventricle, and JQ1 treatment markedly reduced the fibrosis in both regions (Figure 3A–D). Quantitative analysis revealed that the fibrosis area was significantly increased in both the atria and ventricles in the Ang II-treated group, and was significantly reduced by JQ1 treatment, indicating that JQ1 suppresses AngII-induced fibrotic remodeling in atrial and ventricular myocardium.

### 2.4. JQ1 Modulates Fibrosis-Related Gene Expression in Atrial Tissue

**Figure 4:**
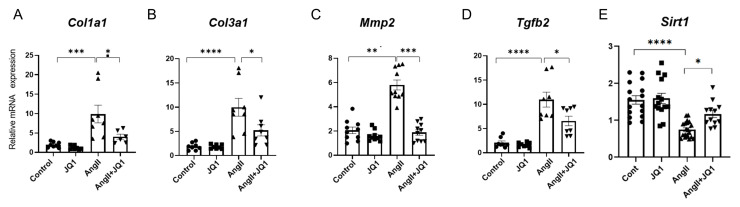
Gene expression analysis in atrial tissue by real-time RT-PCR. mRNA levels of fibrosis and remodeling-related genes: (**A**) *Col1a1*, (**B**) *Col3a1*, (**C**) *Mmp2*, (**D**) *Tgfb2*, and (**E**) *Sirt1*. Expression was normalized to *18S rRNA*. Data shown as mean ± SEM (*n* = 4–8 measurements per group; each measured in duplicate). * *p* < 0.05, ** *p* < 0.005, *** *p* < 0.0005, **** *p* < 0.0001.

To elucidate the molecular mechanisms underlying the antifibrotic effects of JQ1, mRNA expression levels of fibrosis-related genes were measured by RT-PCR in LA tissue. AngII significantly upregulated expression of *Col1a1*, *Col3a1*, *Tgfb2*, and *Mmp2*; all of which were significantly downregulated by JQ1 treatment (Figure 4A–D). Expression of *Sirt1*, a gene involved in cellular metabolism and stress resistance, was suppressed by AngII and restored by JQ1 (Figure 4E). These results suggest that JQ1 attenuates fibrotic remodeling by modulating the transcription of extracellular matrix and regulatory genes.

### 2.5. JQ1 Regulates AF-Related Protein Expression in Cardiac Tissue

**Figure 5:**
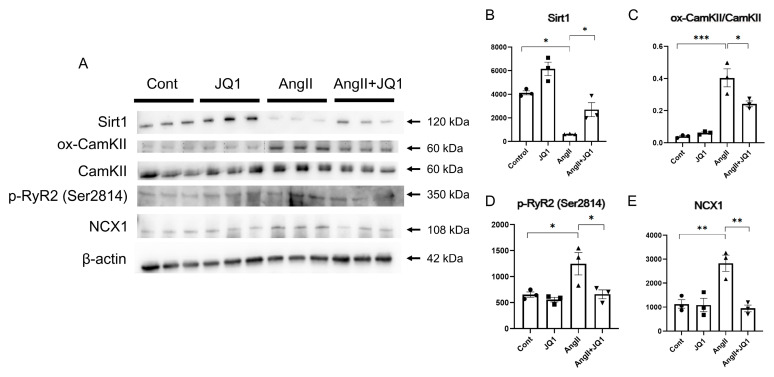
Protein expression analysis in heart tissue by Western blotting. (**A**) Representative Western blot images for SIRT1, ox-CaMKII, CaMKII, p-RyR2 (Ser2814), NCX1, and β-actin. (**B**–**E**) Densitometric quantification of each protein. Values are shown as relative intensity normalized to β-actin (*n* = 3 samples per group). * *p* < 0.05, ** *p* < 0.005, *** *p* < 0.0005.

Western blot analysis of heart tissue revealed that protein level of SIRT1 was significantly reduced in the AngII group and restored by JQ1 treatment (Figure 5A,B). Conversely, expression of oxidative CaMKII (ox-CaMKII), phosphorylated RyR2 at Ser2814 (p-RyR2), and NCX1 was elevated in AngII-treated mice and significantly suppressed by JQ1 (Figure 5C–E). These results suggest that JQ1 modulates key regulators of calcium handling and myocardial remodeling, contributing to reduced AF susceptibility.

### 2.6. JQ1 Attenuates AngII-Induced Abnormal Intracellular Ca^2+^ Handling in HL-1 Cells

**Figure 6:**
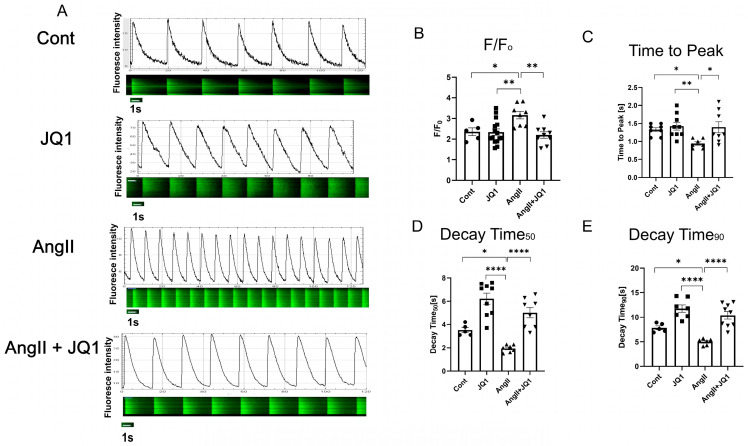
Intracellular Ca^2+^ handling in HL-1 cells. (**A**) Representative confocal line scan and fluorescence traces of spontaneous Ca^2+^ waves. (**B**–**E**) Quantification of: (**B**) Amplitude (F/F_0_), (**C**) TP, (**D**) 50% DT, and (**E**) 90% DT. Data are expressed as mean ± SEM (*n* = 5–12 measurements per group). * *p* < 0.05, ** *p* < 0.005, **** *p* < 0.0001.

Calcium imaging in HL-1 atrial myocytes showed that AngII significantly increased diastolic Ca^2+^ amplitude (F/F_0_), which was normalized by JQ1 treatment (Figure 6A,B). AngII also shortened both time to peak (TP) and decay time (DT) of the Ca^2+^ transient, consistent with dysregulated calcium cycling. These parameters were significantly improved by JQ1 administration (Figure 6C,D). These findings indicate that JQ1 mitigates intracellular Ca^2+^ dysregulation induced by AngII.

### 2.7. JQ1 Normalizes AngII-Induced Prolongation of APD in HL-1 Cells

**Figure 7:**
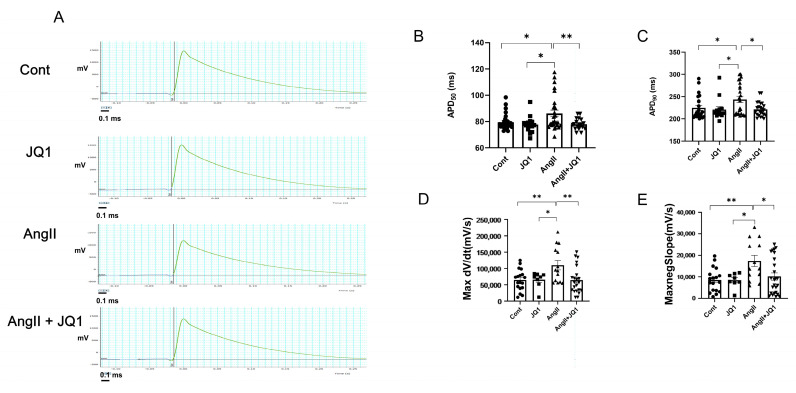
Electrophysiological characteristics of HL-1 cells using MEA. (**A**) Representative extracellular waveform recordings. (**B**–**E**) Quantification of: (**B**) APD_50_; (**C**) APD_90_; (**D**) max dV/dt; and (**E**) −max dV/dt. Data are expressed as mean ± SEM (*n* = 8–25 measurements per group). * *p* < 0.05, ** *p* < 0.005.

Electrophysiological recordings using a multi-electrode array (MEA) system demonstrated that AngII significantly prolonged APD in HL-1 cells, while JQ1 restored APD to levels comparable with controls (Figure 7A–C). Additionally, both maximal upstroke velocity (Max dV/dt) and downstroke velocity (−Max dV/dt) were increased in the AngII group and normalized by JQ1 (Figure 7D,E). These results suggest that JQ1 prevents AngII-induced electrophysiological remodeling in atrial-like cardiomyocytes.

### 2.8. JQ1 Preserves Mitochondrial Function in AngII-Treated HL-1 Cells

**Figure 8:**
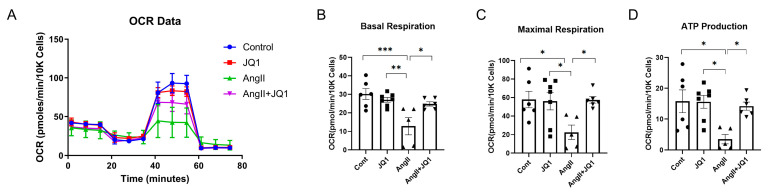
Mitochondrial respiration analysis in HL-1 cells. (**A**) Oxygen consumption rate (OCR) curves measured by Seahorse XF Mito Stress Test (colors: blue = control; red = JQ1; green = AngII; purple = AngII+JQ1). (**B**–**D**) Quantification of mitochondrial respiratory parameters: (**B**) Basal respiration; (**C**) Maximal respiration; and (**D**) ATP production (pmol/min/50K cells). Values presented as mean ± SEM (*n* = 5–7 measurements per group). * *p* < 0.05, ** *p* < 0.005, *** *p* < 0.0005.

Mitochondrial stress test revealed that basal respiration and maximal respiratory capacity were significantly impaired in AngII-treated HL-1 cells, and both were restored by JQ1 treatment (Figure 8A–C). JQ1 also reversed the AngII-induced reduction in ATP production (Figure 8D). These findings indicate that JQ1 protects mitochondrial metabolic function in atrial cardiomyocytes under AngII-induced stress.

## 3. Discussion

In this study, we demonstrated that the non-selective BET inhibitor JQ1 effectively attenuates AngII–induced vulnerability to AF in a mice model. Treatment with JQ1 significantly mitigated hallmark pathological features of AF, including LA enlargement, diastolic dysfunction, and myocardial fibrosis in both atrial and ventricular tissue. Furthermore, JQ1 reversed electrical remodeling, as reflected by normalization of the AERP and restoration of intracellular calcium dynamics, and improved mitochondrial function in atrial cardiomyocytes. Collectively, these findings suggest that BET inhibition may serve as a novel therapeutic strategy targeting multiple pathophysiological aspects of AF.

A key molecular finding was the restoration of Sirt1 expression in atrial tissue following JQ1 administration. Sirtuin 1 (SIRT1), a NAD^+^-dependent histone deacetylase, regulates various cellular processes, including metabolism, oxidative stress responses, and transcriptional repression [11]. Previous studies have reported that SIRT1 plays a critical role in suppressing extracellular matrix deposition and fibrosis through inhibition of the TGF-β signaling pathway [12,13,14,15]. Furthermore, SIRT1 overexpression has been shown to attenuate atrial structural remodeling and fibrosis in animal models [16]. These observations support the hypothesis that BET inhibition enhances SIRT1 activity and suppresses the TGF-β pathway, thereby contributing to the prevention of atrial fibrosis.

In line with this, our in vitro data demonstrated that Ang II impaired mitochondrial respiration in HL-1 atrial cardiomyocytes, while JQ1 treatment preserved mitochondrial function. Given that SIRT1 supports mitochondrial homeostasis by promoting biogenesis and attenuating oxidative stress, our results suggest that BET inhibition may protect mitochondrial integrity via SIRT1-related mechanisms. Mitochondrial dysfunction is increasingly recognized as a contributor to AF development, particularly through its role in promoting oxidative injury and metabolic impairment. Therefore, the observed restoration of mitochondrial respiration and ATP production by JQ1 may represent a novel antiarrhythmic mechanism mediated through preservation of cellular energy metabolism.

The role of BET proteins—particularly BRD4—as global transcriptional regulators is well established in cancer and inflammatory diseases [17,18,19]. Several BET inhibitors, including JQ1, are currently undergoing clinical evaluation in oncology [20], and recent studies suggest a potential role for BRD4 in cardiovascular diseases [7,8,9,10,21,22,23,24]. In the present study we demonstrated that JQ1 inhibited not only atrial fibrosis but also ventricular fibrosis induced by AngII, and improved diastolic function indicated as the E/A. In the context of AF, BRD4 has been implicated in promoting atrial fibrosis by enhancing extracellular matrix production and fibroblast proliferation in response to Ang II stimulation [25]. Taken together with our findings, these data support the potential of BET inhibition as a therapeutic strategy not only for AF but also for other cardiovascular disorders characterized by structural remodeling and inflammation [26].

This study has several limitations. First, the effects of JQ1 were evaluated exclusively in a mice Ang II–induced AF model and in HL-1 atrial cardiomyocytes. Thus, the findings may not be generalizable to other experimental or clinical models of AF. Further validation in diverse in vivo and in vitro systems is necessary to establish the broader relevance and translational potential of BET inhibition. Second, the present study focused on short-term treatment outcomes. The long-term efficacy and safety of JQ1 in preventing atrial remodeling and arrhythmogenesis remain unknown. Third, although our data suggest that JQ1 exerts its effects through epigenetic modulation, particularly involving SIRT1 and mitochondrial preservation, the underlying molecular pathways remain incompletely defined. Given the complex regulatory roles of BET proteins, further mechanistic studies are warranted to elucidate the downstream targets contributing to structural and electrical remodeling in AF. Fourth, Western blotting was conducted using whole heart tissue because the amount of protein obtained from atrial tissue alone was insufficient. Therefore, the results may not fully reflect the molecular changes occurring in the atria during AF. Finally, Western blot analysis was performed using whole-heart tissue rather than isolated atrial samples due to the limited amount of atrial tissue obtainable from each mouse. As a result, the detected protein signals may not be strictly atrial-specific, representing a technical constraint that could have partially masked atrial-specific molecular changes. Future studies employing atrial-enriched or region-specific analyses will be necessary to confirm these findings. 

## 4. Materials and Methods

### 4.1. Animal Ethics and Welfare Considerations

All animal experiments were conducted in accordance with the Guide for the Care and Use of Laboratory Animals (U.S. National Institutes of Health), the Animal Experiment Regulations of the University of Tsukuba, and the Basic Guidelines for the Proper Conduct of Animal Experiments in Academic Research Institutions issued by the Ministry of Education, Culture, Sports, Science and Technology of Japan. All procedures were approved by the Animal Experiment Committee of the University of Tsukuba (Approval No: 24-309) and were carried out at the university’s Laboratory Animal Resource Center. All personnel involved in animal handling were certified through the university’s mandatory training program in laboratory animal care and welfare. The design, conduct, and reporting of the animal experiments adhered to the ARRIVE guidelines (Animal Research: Reporting of In Vivo Experiments).

Animals were monitored once or twice daily for clinical signs of distress, including reduced mobility, weight loss exceeding 20%, labored breathing, or inability to access food or water. Humane endpoints were predefined prior to the start of the study. According to these criteria, animals exhibiting severe signs of distress would have been humanely euthanized and excluded from further analysis. However, no animals met the exclusion criteria during the study, and no euthanasia for humane endpoints was required. No other inclusion or exclusion criteria for animals or data points were applied.

Anesthesia was induced via intraperitoneal injection of ketamine (50 mg/kg) and xylazine (7.5 mg/kg). Analgesia was administered as needed to minimize pain and discomfort. Euthanasia was performed either by gradual-fill exposure to carbon dioxide (CO_2_) or by exsanguination under deep anesthesia, in accordance with the AVMA Guidelines for the Euthanasia of Animals. All efforts were made to minimize suffering and ensure ethical treatment throughout the study.

### 4.2. Experimental Design

This study involved both in vivo and in vitro experiments. In vivo experiments were conducted using a mice AF model induced by continuous AngII infusion, while in vitro experiments utilized HL-1 atrial cardiomyocytes. The outcome measures assessed included AF induction rate, AF duration, echocardiographic parameters, fibrotic area, expression of fibrosis-related and calcium-handling-related molecules, cellular electrophysiological parameters, and mitochondrial function.

A total of 75 twelve-week-old male C57BL/6 mice (25–30 g; Japan Charles River, Yokohama, Kanagawa, Japan) were used. All animals were housed in a temperature-controlled facility under a 12h light/dark cycle, with free access to tap water and standard chow. Mice were acclimatized for at least 1 week prior to any interventions, and were randomly assigned to one of four groups using a non-systematic allocation method: Control, JQ1 (50 mg/kg/day), AngII (1 μg/kg/min) + Vehicle, and AngII + JQ1. Each individual mouse was considered an experimental unit. The study period lasted 15 days. AngII (1 μg/kg/h) was administered continuously for 14 days via subcutaneously implanted osmotic minipumps (Alzet Model 2002, Cupertino, CA, USA). Control mice received normal saline through identical pumps. JQ1 was suspended in a vehicle composed of 10% dimethyl sulfoxide (DMSO) and 10% 2-hydroxypropyl-β-cyclodextrin, and administered intraperitoneally once daily at a dose of 50 mg/kg for 14 consecutive days. The dosage and administration route of AngII and JQ1 were based on previously published studies that demonstrated effective cardiac remodeling and anti-fibrotic effects, respectively. The timeline and endpoints were selected to evaluate the effects of treatment on cardiac function, fibrosis, and arrhythmia susceptibility in a controlled manner. Mice in the Control and AngII groups received equivalent volumes of vehicle as sham treatment. All pump implantations were performed under anesthesia induced by intraperitoneal injection of ketamine and xylazine. During the study, three mice died unexpectedly (one from the Control group and two from the AngII group) and were excluded from the final analysis. Following the 14-day treatment period, 42 mice (Control: *n* = 11; JQ1: *n* = 9; AngII: *n* = 12; AngII + JQ1: *n* = 10) underwent electrophysiological studies, including AF induction. The sample size (>8 per group) was based on previous AF induction studies using similar models. AF duration was used as the primary outcome measure for sample size estimation. The remaining 30 mice were used for direct cardiac tissue collection without undergoing electrophysiological testing, and the tissues were subjected to histological and molecular analyses. To minimize potential confounders, drug administration, electrophysiological assessments, and tissue collection were conducted in a randomized order. Mice were assigned identification numbers to conceal group allocation. AF induction and echocardiographic measurements were performed by two dedicated investigators blinded to group assignment.

### 4.3. Electrophysiological Study and AF Induction 

In vivo electrophysiological study was performed as previously described [27,28,29]. Mice were anesthetized with intraperitoneal injections of ketamine (50 mg/kg) and xylazine (7.5 mg/kg). A 1.2-Fr quadripolar catheter with 2 mm interelectrode spacing (Unique Medical, Tokyo, Japan) was inserted into the right atrium via the cervical vein. The catheter tip was positioned to optimize intra-atrial electrogram amplitude relative to the intraventricular signal. AERPs were measured using a programmable stimulator (SEN-7203; Nihon Kohden, Tokyo, Japan). A stimulus train of eight basic stimuli (S1 × 8) was followed by a single extrastimulus (S2), delivered at 5 ms decrements. AERP was defined as the shortest S1–S2 interval that consistently resulted in atrial capture. To induce AF, burst pacing was applied using the electrode catheter with the following parameters: 6 V amplitude, 20 ms cycle length, 6 ms pulse duration, and 30 s duration per burst. Pacing was repeated five times per mouse, and the AF induction rate was calculated as the number of induced episodes divided by the number of attempts. AF duration was defined as the interval from onset to spontaneous termination of arrhythmia.

### 4.4. Echocardiography 

Transthoracic echocardiography was performed using the Vevo 2100 imaging system (FUJIFILM VisualSonics, Toronto, ON, Canada) equipped with an MS-400 transducer. Mice were anesthetized with 1% isoflurane during the examination. The following parameters were measured using Vevo Strain software (version 5.7.1): LVDd, LVDs, LVEF, LAD, and E/A.

### 4.5. HL-1 Cell Culture

HL-1 atrial myocytes were cultured as described previously [28]. HL-1 cells were maintained in Claycomb medium supplemented with 10% fetal bovine serum (FBS), 100 U/mL penicillin, 100 µg/mL streptomycin, 2 mM L-glutamine, 100 μM norepinephrine, and 30 mM L-ascorbic acid at 37 °C in a humidified incubator containing 5% CO_2_. For JQ1 treatment studies, HL-1 cells were seeded into 6-well plates precoated with 0.02% (*w*/*v*) gelatin containing 5 μg/mL fibronectin. Cells were stimulated with AngII (1 μM) for 24 hours either with (AngII + JQ1 group) or without (AngII group) JQ1 (500 nM). Cells were subsequently harvested for further analysis.

### 4.6. Calcium Imaging of HL-1 Cells

Calcium imaging of HL-1 cells was performed as previously described [27,28,30,31]. HL-1 cells were plated onto gelatin/fibronectin-coated glass-bottom dishes (24 × 32 mm, No. 1-S; Matsunami). After attachment, cells were loaded with 5 μM Fluo-4 AM (Invitrogen, Carlsbad, CA, USA) dissolved in 20% Pluronic F-127 in DMSO and diluted in Tyrode’s solution (140 mM NaCl, 5 mM KCl, 5 mM HEPES, 2 mM NaH_2_PO_4_, 1 mM MgCl_2_, 2 mM CaCl_2_, and 10 mM glucose; pH 7.4). HL-1 cells were subjected to serum starvation before AngII treatment and were subsequently incubated for 10 min at 37°C, washed, and equilibrated for an additional 10 min before imaging. Single cells were imaged with a laser scanning confocal microscope, with the scan line positioned through the nucleus. The frequency of abnormal Ca^2+^ waves was defined as the proportion of cells displaying spontaneous irregular Ca^2+^ transients among the total number of analyzed cells. The values of F/F0, TP, DT_50_, and DT_90_ were measured using ImageJ software (version 1.53v, National Institutes of Health, Bethesda, MD, USA).

### 4.7. APD Measurement in HL-1 Cells

APD was measured using a MEA system as previously described [28]. HL-1 cells were seeded onto precoated 6-well MEA dishes (6wellMEA200/30iR-Ti-tcr; Multi Channel Systems MCS GmbH, Reutlingen, Germany). After stabilization at 37 °C, the cells were stimulated using bipolar pulses (+/−7500 mV) at 3 Hz for 30 cycles using the MEA2100 platform (Multi Channel Systems). Data were acquired using LabChart software (version 8, ADInstruments, Colorado Springs, CO, USA) and filtered at 100 Hz to reduce electrical noise. APD values were extracted from the recorded waveforms. 

### 4.8. Histological Analysis 

Cardiac tissues (left atrium and left ventricle) were fixed in 4% paraformaldehyde, embedded in paraffin, and sectioned at 4 μm thickness. Sections were stained with Masson’s trichrome. Fibrosis was quantified by calculating the ratio of collagen-stained (aniline blue) area to total tissue area using images obtained under an optical microscope (BZX710; Keyence, Osaka, Japan). Fibrotic areas were quantified using ImageJ software. 

### 4.9. Real-Time Quantitative Polymerase Chain Reaction (PCR)

Total RNA was extracted from atrial tissue using the RNeasy Mini Kit (Qiagen, Valencia, CA, USA). Reverse transcription was carried out with 1 μg of RNA using the High-Capacity cDNA Reverse Transcription Kit (Thermo Fisher Scientific, Waltham, MA, USA) as previously described2 [29]. Quantitative real-time PCR was performed on an ABI Prism 7500 FAST system (Applied Biosystems, Foster City, CA, USA) using PrimeTime Gene Expression Master Mix (Integrated DNA Technologies, Coralville, IA, USA). The following primers were used: Col1a1 (Mm.PT.58.7562513), Col3a1(Mm.PT.58.13848686), Mmp2 (Mm.PT.58.9606100), Tgfb2 (Mm.PT.58.14105470), and Sirt1 (Mm.PT.58.7263242). Gene expression was normalized to the housekeeping gene 18S rRNA (4319413E; Thermo Fisher Scientific).

### 4.10. Western Blotting 

Western blotting was performed using heart tissue extracts, as previously described [27,28,32]. Briefly, heart tissue was homogenized in PRO-PREP protein extraction solution (iNtRON Biotechnology Inc., Seongnam-si, Gyeonggi-do, Republic of Korea). A total of 10 μg of protein was separated by SDS-PAGE using polyacrylamide gels (Bio-Rad Laboratories, Hercules, CA, USA), followed by semi-dry electrotransfer to PVDF membranes. Membranes were incubated overnight at 4°C with the following primary antibodies: phosphorylated RyR2 at Ser2814 (p-RyR2; A010-31AP, Badrilla, Leeds, UK), oxidized CaMKII (ox-CaMKII; 07-1387, Merck, Darmstadt, Germany), total CaMKII (3362, Cell Signaling Technology, Danvers, MA, USA), SIRT1 (sc-19857, Santa Cruz Biotechnology, Dallas, TX, USA), NCX1 (ab177952, Abcam, Cambridge, UK), and β-actin (4967, Cell Signaling Technology, Danvers, MA, USA). After washing, membranes were incubated for 1 hour at room temperature with appropriate secondary antibodies: HRP-conjugated goat anti-rabbit IgG (ab6721, Abcam) or HRP-conjugated rabbit anti-mouse IgG (ab97046, Abcam). Protein bands were visualized using an enhanced chemiluminescence detection system (ECL Prime Western Blotting Detection Reagent; GE Healthcare, Chicago, IL, USA).

### 4.11. Mitochondrial Stress Test in HL-1 Cells

Mitochondrial function in HL-1 cells was assessed using the Seahorse XF Analyzer (Agilent Technologies, Santa Clara, CA, USA), as described previously [32]. HL-1 cells were seeded in precoated XF24 microplates and maintained using standard culture conditions. Prior to the assay, the culture medium was replaced with Seahorse XF Cell Mito Stress Test Assay Medium (XF DMEM, pH 7.4), supplemented with 10 mM glucose, 1 mM sodium pyruvate, and 2 mM L-glutamine. The Cell Mito Stress Test was conducted using sequential injections of 1 μM oligomycin, 1.25 μM FCCP, and a mixture of 0.5 μM rotenone and antimycin A to evaluate the OCR. ATP production was assessed by inhibition of ATP synthase with oligomycin. FCCP, a protonophore that collapses the mitochondrial membrane potential, was used to assess maximal respiration. Rotenone and antimycin A inhibit complexes I and III, respectively, allowing assessment of non-mitochondrial respiration. The results of basal respiration, maximal respiration, and ATP production were measured using Wave Desktop Software (version 2.6.1, Agilent Technologies).

### 4.12. Statistical Analysis

All data are presented as mean ± standard error of the mean (SEM). The normality of data distribution was assessed using the Shapiro–Wilk test. For comparisons among multiple groups, one-way analysis of variance (ANOVA) was performed, followed by Tukey’s post hoc test for normally distributed data. If the assumptions of normality were not met, appropriate non-parametric tests (e.g., Kruskal–Wallis test with Dunn’s post hoc test) were applied. A *p*-value of <0.05 was considered statistically significant. All statistical analyses were performed using GraphPad Prism (version 9; GraphPad Software, San Diego, CA, USA).

## 5. Conclusions

In conclusion, our study demonstrates that BET inhibition by JQ1 suppresses atrial structural and electrical remodeling through the restoration of SIRT1 expression, preservation of mitochondrial function, and normalization of calcium handling. These findings highlight BET inhibition as a promising therapeutic approach for AF. Future preclinical and translational studies are needed to further explore its clinical applicability and mechanistic underpinnings.

## Data Availability

All data generated or analyzed during this study are included in this published article.

## Abbreviations

AERPs	atrial effective refractory periods
AF	atrial fibrillation
AngII	angiotensin II
APD	action potential duration
ATP	adenosine triphosphate
BD1	bromodomain 1
BD2	bromodomain 2
BET	bromodomain and extraterminal domain
BRD2	bromodomain-containing protein 2
BRD3	bromodomain-containing protein 3
BRD4	bromodomain-containing protein 4
BRDT	bromodomain testis-specific protein
CamKII	calcium/calmodulin-dependent protein kinase II
Col1a1	collagen type I alpha I chain
Col3a1	collagen type III alpha I chain
DT	decay time
E/A	early-to-atrial filling velocity ratio
LA	left atrial
LAD	left atrial-diameter
LV	left ventricular
LVDd	left ventricular end-diastolic diameter
LVDs	left ventricular end-systolic diameter
LVEF	left ventricular ejection fraction
MEA	multi-electrode array
MMP2	matrix metalloproteinase-2
NAD^+^	nicotinamide adenine dinucleotide
NCX1	sodium/calcium exchanger
OCR	oxygen consumption rate
RyR2	ryanodine receptors 2
Sirt1	sirtuin 1
Tgfb2	transforming growth factor beta-2
TP	time to peak
